# Production of butyrate from lysine and the Amadori product fructoselysine by a human gut commensal

**DOI:** 10.1038/ncomms10062

**Published:** 2015-12-01

**Authors:** Thi Phuong Nam Bui, Jarmo Ritari, Sjef Boeren, Pieter de Waard, Caroline M. Plugge, Willem M. de Vos

**Affiliations:** 1 Laboratory of Microbiology, Wageningen University, Dreijenplein 10, 6703 HB Wageningen, The Netherlands; 2 Department of Basic Veterinary Medicine, University of Helsinki, Agnes Sjöberginkatu 2, FIN-00790 Helsinki, Finland; 3 Laboratory of Biochemistry, Wageningen University, Dreijenlaan 3, 6703 HA Wageningen, The Netherlands; 4 Wageningen NMR Centre, Dreijenlaan 3, 6703 HA Wageningen, The Netherlands; 5 Department of Bacteriology and Immunology, Haartmaninkatu 3, University of Helsinki, FIN-0014 Helsinki, Finland

## Abstract

Human intestinal bacteria produce butyrate, which has signalling properties and can be used as energy source by enterocytes thus influencing colonic health. However, the pathways and the identity of bacteria involved in this process remain unclear. Here we describe the isolation from the human intestine of *Intestinimonas* strain AF211, a bacterium that can convert lysine stoichiometrically into butyrate and acetate when grown in a synthetic medium. *Intestinimonas* AF211 also converts the Amadori product fructoselysine, which is abundantly formed in heated foods via the Maillard reaction, into butyrate. The butyrogenic pathway includes a specific CoA transferase that is overproduced during growth on lysine. Bacteria related to *Intestinimonas* AF211 as well as the genetic coding capacity for fructoselysine conversion are abundantly present in colonic samples from some healthy human subjects. Our results indicate that protein can serve as a source of butyrate in the human colon, and its conversion by *Intestinimonas* AF211 and related butyrogens may protect the host from the undesired side effects of Amadori reaction products.

## Abstract (web summary)

Bacterial production of butyrate in the gut is associated with a healthy colon. Here the authors isolate an *Intestinimonas* strain from the human gut that can produce butyrate from lysine and fructoselysine, a potentially harmful compound formed in heated foods.

## Graphical abstract

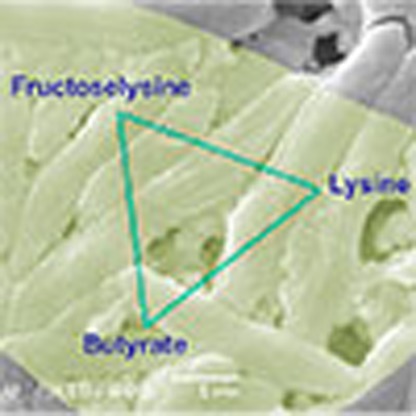

Our intestinal tract contains trillions of microbes that impact our health^1^. The metabolic capacity of these microbes is enormous^2^ and includes the production of short-chain fatty acids, such as butyrate that serves as a major source of energy for the intestinal enterocytes and signals to the host^3^. Butyrogenic gut bacteria belong to Lachnospiraceae and Ruminococcaceae^4^, and recent studies showed an inverse correlation between these butyrogenic Firmicutes and inflammatory bowel disease, type 2 diabetes and various other disorders^5^,^6^. Moreover, there is considerable interest in these butyrogens, since butyrate reduces proinflammatory signals^3^ and has a protective role in maintaining a healthy colon^7^,^8^. Thus, it is of paramount importance to identify the microbes that are capable of producing butyrate and understand the mechanisms underlying its production.

Most butyrate produced in the human intestine is assumed to derive from carbohydrates, while some specialized bacteria have been found that convert lactate plus acetate into butyrate^9^,^10^. All of these produce butyrate via the acetyl-CoA pathway^11^ (converting acetyl-CoA to butyrate) that involves a complex cascade of reactions, in which butyrate CoA transferase (But) or butyrate kinase (Buk) are key enzymes. In a recent (meta)genome-based study, it was predicted that the But-mediated route was 10-fold more abundant than that mediated by Buk, while these two enzymes showed high diversity among butyrogens^12^. Three other routes for butyrate synthesis, the glutamate, succinate and lysine pathways, have been described. On the basis of the distribution of the genes in the intestinal metagenome libraries, the acetyl-CoA pathway was the most prevalent followed by the lysine pathway. Yet, no intestinal bacterium has been reported that contained all genes coding for butyrogenesis from this amino acid^13^.

In addition to carbohydrates, proteins are crucial dietary compounds that are receiving increased attention to their use in special diets. Proteolytic enzymes in the upper intestinal tract generate amino acids that are rapidly taken up by the host, but also may reach the colon, partly depending on the intake. Moreover, protein recycling in the colon has been described, adding to the interest in colonic amino-acid fermentations. Amadori products (fructosamines), formed via non-enzymatic reactions between reducing sugars and free amino groups of proteins during the food heating process, are of significant interest as they have shown to be associated with the ageing process and several chronic diseases^14^. Fructoselysine is a key intermediate in the formation of advanced glycation endproducts (AGEs)^14^ and not yet any organism has been known to convert fructoselysine into butyrate. Only a few microbes have been reported to metabolize lysine into butyrate, and they derive from ecosystems other than the gut. A lysine degradation pathway was described over 50 years ago in *Clostridium sticklandii* isolated from black mud^15^. More recently, this pathway was detected in *Clostridium subterminale* SB4 isolated from sewage and *Fusobacterium nucleatum* isolated from the oral cavity, and eight genes encoding a lysine fermentation pathway were identified from the genome of *F. nucleatum*^16^. Several enzymes involved in butyrogenesis from lysine have been biochemically characterized^17^,^18^,^19^,^20^, but so far no intestinal microorganism has been found to contain the entire pathway in spite of the predicted presence of the lysine fermentation pathway in the human intestine^13^,^16^,^17^,^18^. Here we describe the butyrogenic conversion of lysine and the Amadori product fructoselysine by a novel *Intestinimonas* strain from a healthy volunteer and characterize its butyrate synthesis pathway by using a combined physiological, biochemical and proteogenomic approach.

## Results

### Lysine degradation by *Intestinimonas* AF211

In a search for new butyrogens, strain AF211 was isolated from the stool of a healthy subject using a mineral bicarbonate-buffered medium with lactate and acetate, under strict anoxic conditions (butyrate was found as the main end product). Strain AF211 contained two copies of the 16S rRNA gene that differed in a single nucleotide, and subsequent phylogenetic analysis showed that this strain belonged to clostridial cluster IV (Lachnospiraceae) of the Firmicutes phylum. Further analysis showed that this 16S rRNA sequence was highly similar to that of the mouse intestinal isolate *Intestinimonas butyriciproducens* DSM26588^T^ (ref. 21), indicating that the newly isolated strain AF211 belongs to the genus *Intestinimonas* (Supplementary Fig. 1). However, strain *Intestinimonas* AF211 was a human isolate and showed physiological differences with *I. butyriciproducens* from mouse (to be reported elsewhere). Butyrate was found to be a major metabolite from all substrates where growth was observed (Supplementary Table 1). *Intestinimonas* AF211 hardly grew on glucose in mineral medium (generation time of 88 days), but its growth rate tripled in the presence of acetate and yeast extract. In media containing 20 mM glucose with 20 mM acetate and 2% yeast extract, *Intestinimonas* AF211 produced up to 4.4 mM butyrate and trace amounts of lactate and ethanol (2-week incubation). Remarkably, the strain grew much better (generation time of 7.5 h) in L-lysine but not in any other natural amino acids or D-lysine (Supplementary Table 1).

**Figure 1:**
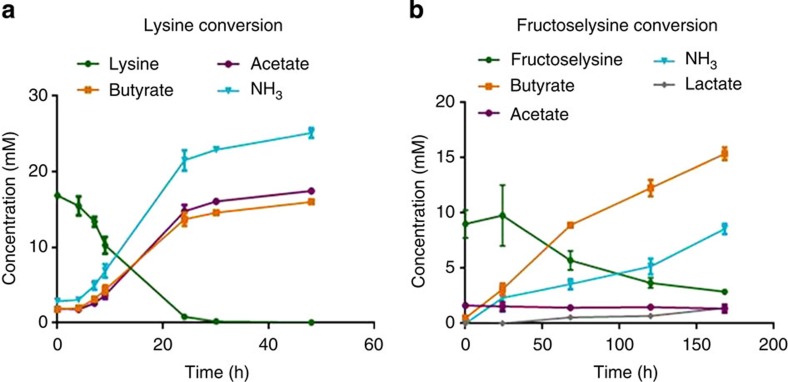
L-lysine and fructoselysine conversion by strain *Intestinimonas* AF211 throughout time. Carbon recovery was 87% and 101% for the lysine (**a**) and fructoselysine (**b**) utilization, respectively. Exact concentrations of substrates and products and optical density values are included in Supplementary Table 1. Values are mean of biological duplicates. Error bars indicate s.d.

To study the capacity to convert lysine into butyrate, *Intestinimonas* AF211 was grown in bicarbonate-buffered medium containing L-lysine as the sole carbon and energy source. A total of 16.8±0.4 mM lysine was converted into 14.2±0.6 mM butyrate, 15.6±0.7 mM acetate and 22.1±0.5 mM NH_3_ when the cells reached the stationary phase after 2 days at 37 °C (Fig. 1a). This suggests that part of the released ammonia is sequestered into proteins during anabolism of *Intestinimonas* AF211. Hence, we propose the fermentation reaction as: C_6_H_14_O_2_N_2_+2H_2_O→C_4_H_8_O_2_+C_2_H_4_O_2_+2NH_3_. Clearly, *Intestinimonas* AF211 is capable of growing in defined media with L-lysine as sole carbon and energy source with a maximum growth rate of 0.1 h^−1^.

### Growth of *Intestinimonas* AF211 on fructoselysine

**Figure 2:**
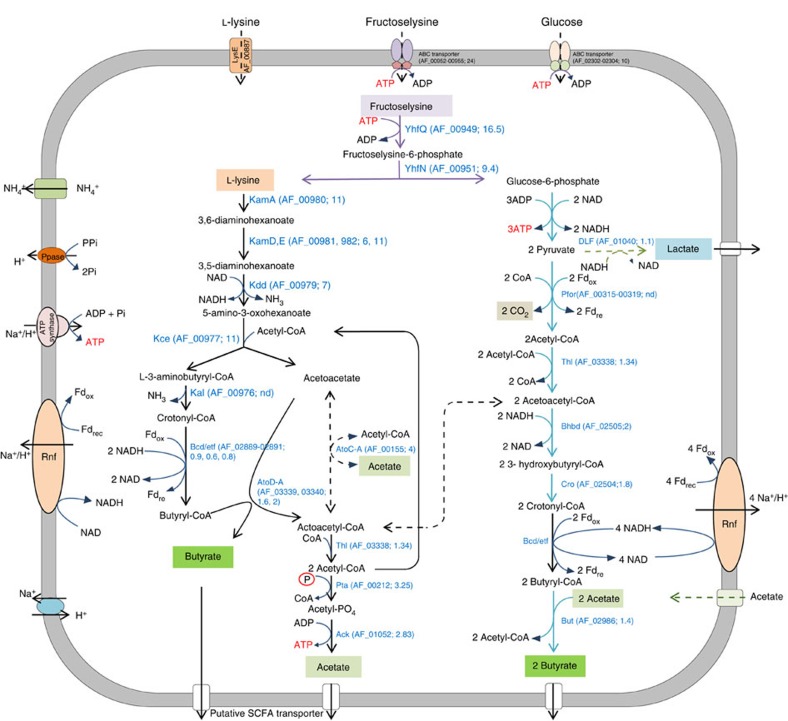
Model for fructoselysine metabolism in *Intestinimonas* AF211. The locus tag and fold induction of the proteins based on the proteomic data are indicated in the brackets. Fd, ferredoxin; ND, not detected; Ppase, pyrophosphatase; Rnf, proton pumping Rnf cluster. According to our model, fructoselysine is taken up by ABC transporter (AF_00952-00955), which is phosphorylated by fructoselysine kinase (AF_00949) to form fructoselysine-6-phosphate that is subsequently cleaved by fructoseamine deglycase (AF_00951) into lysine and glucose-6-phosphate (indicated by the purple arrows). Lysine is then degraded via the lysine pathway (black arrows) while glucose-6-phosphate is metabolized via glycolysis and the acetyl-CoA pathway (blue arrows) as described in the text (Supplementary Fig. 4). There are various links between the lysine and acetyl-CoA pathways and the one at the level of acetoacetyl-CoA, involving acetate to generate acetoacetate and acetyl-CoA, is indicated (dashed black arrows). For simplicity, only the key reactions are shown and other links or the conversion of acetyl-CoA formed via acetyl-CoA pathway to acetate have been omitted. Fructoselysine, lysine, butyrate, acetate, lactate and CO_2_ are indicated in different colours highlighting their distinctive positions in the pathways. ATP is in red to indicate reactions that either require or generate energy via substrate-level phosphorylation and electron transport chain. Lactate formation is dependent on the amount of exogenous acetate and redox state (hence both lactate formation and acetate uptake are indicated by dashed green arrows). Therefore, the overall fructoselysine stoichiometry depends on the presence or absence of exogenous acetate as well as environmental conditions and activity of biosynthetic pathways. In the presence of acetate (such as in the human colon), fructoselysine is converted in approximately three butyrate (two butyrate are formed via acetyl-CoA pathway and one butyrate through the lysine pathway), while no lactate is produced; however, when no exogenous acetate is present, fructoselysine is converted into approximately two butyrate and one lactate (see Supplementary Table 2 for details).

As fructoselysine is a key intermediate in the formation of AGEs, we addressed its degradation by *Intestinimonas* AF211 and found it to be readily metabolized (Fig. 1b; generation time 24 h). Butyrate, NH_3_ and lactate were formed as the main products. The conversion equation is proposed as: C_12_H_24_O_7_N_2_+2 H_2_O→2 C_4_H_8_O_2_+2 NH_3_+1 CO_2_+1 C_3_H_6_O_3_. We anticipate that the fructoselysine pathway includes the simultaneous degradation of both lysine and the sugar moiety in two branches (termed lysine pathway and acetyl-CoA pathway; Fig. 2). Some deviations in the butyrate/fructoselysine ratio were observed (Supplementary Tables 1 and 2). These may be attributed to the different environmental conditions but also be caused by the intracellular balance between the two branches of the fructoselysine pathway (Fig. 2). However, the initial concentration of acetate was predicted to be a major factor affecting the butyrate/fructoselysine ratio. We experimentally verified this and observed that in the presence of extra acetate, fructoselysine was completely converted into butyrate and NH_3_ (Supplementary Table 2) according to the equation: C_12_H_24_O_7_N_2_+C_2_H_4_O_2_+H_2_O→3 C_4_H_8_O_2_+2 NH_3_+2 CO_2_. The growth rate in fructoselysine plus acetate was significantly higher than that in fructoselysine alone (Supplementary Table 2), which could be due to stimulation of butyrate production via external acetate, leading to an increased energy gain by generating a proton motive force via the membrane-associated Rnf complex and consequently, no extra NADH needed for lactate formation (Fig. 2). The influence of acetate on the growth of fructoselysine resembles the human gut environment where acetate is abundantly present^11^. Previously, the conversion of fructoselysine has been reported for a few bacteria, including *Escherichia coli*, which also converts psicoselysine^22^,^23^. However, none of these bacteria are capable of butyrogenesis. To provide further support for the butyrogenic pathway we focused on lysine and glucose degradation by using a combined approach of nuclear magnetic resonance (NMR), proteogenome and enzyme activity assays.

### Elucidation of the lysine degradation pathway using ^13^C-NMR

**Figure 3:**
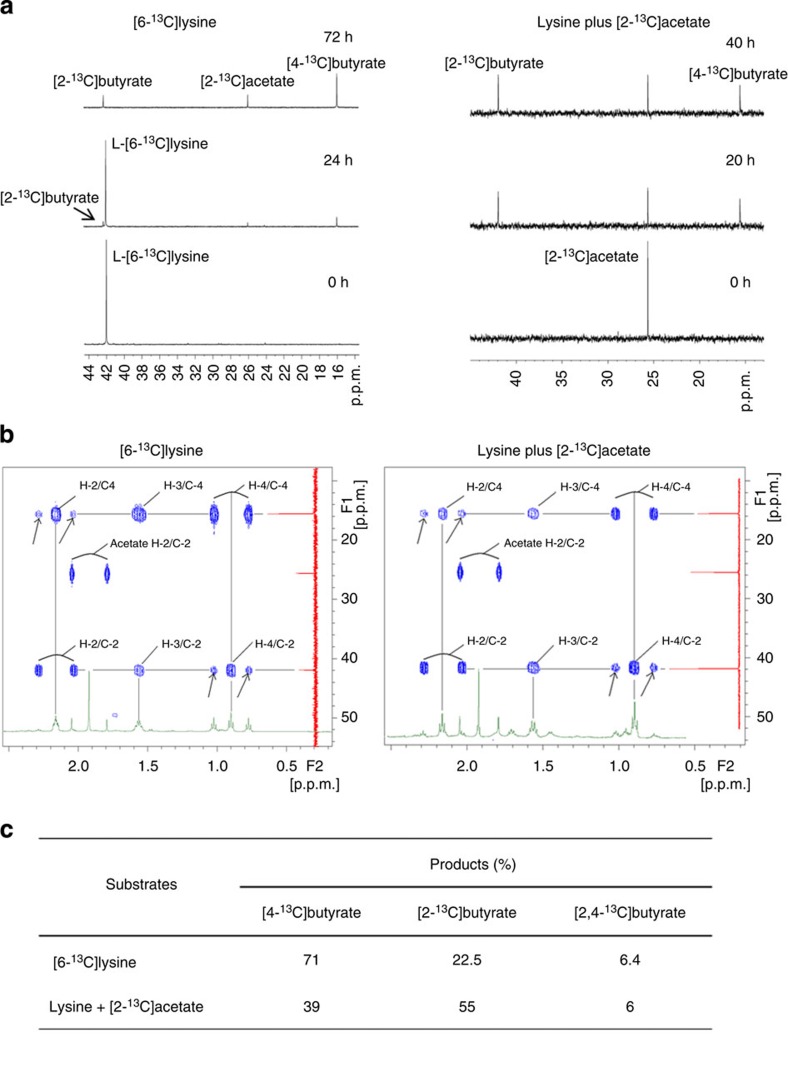
Elucidation of lysine pathway via ^1^H-decoupled ^13^C-NMR spectrum and 2D HMBC spectrum. (**a**) High-resolution ^1^H-decoupled ^13^C-NMR spectra showing L-[6-^13^C]lysine ^13^C-labelled fermentation products. [2-^13^C]butyrate, [2-^13^C]acetate and [4-^13^C]butyrate had a chemical shift of 42.33, 25.99 and 15.95 p.p.m., respectively. (**b**) 2D HMBC spectrum for [6-^13^C]lysine is shown. (**c**) Percentages of labelled butyrate fractions (see Supplementary Figs 2 and 3 for more details).

To elucidate the butyrogenic pathway of *Intestinimonas* AF211, we applied *in vivo*^1^H-decoupled ^13^C-NMR analysis of the culture supernatants of cells grown with L-[2-^13^C]lysine, L-[6-^13^C]lysine and lysine plus [2-^13^C]acetate as described previously^24^. Growth of *Intestinimonas* AF211 on L-[6-^13^C]lysine resulted in its complete conversion into [4-^13^C]butyrate, [2-^13^C]acetate, [2-^13^C]butyrate and [2,4-^13^C]butyrate (Fig. 3a, left). Proton-detected multiple-bond spectroscopy was also performed with the supernatant and based on the Heteronuclear Multiple-Bond Correlations (HMBCs) we could estimate the percentages of [4-^13^C]butyrate, [2-^13^C]butyrate and [2,4-^13^C]butyrate out of all labelled butyrate (Fig. 3b, left). Combination of this quantitative and kinetic analysis showed that at all time points the main product formed from L-[6-^13^C]lysine was [4-^13^C]butyrate (71%; Fig. 3c), indicating that cleavage of lysine occurred between the C2 and C3 residues. Both [4-^13^C]butyrate, [2-^13^C]butyrate and [2,4-^13^C]butyrate were detected in the cells grown in lysine plus [2-^13^C]acetate (Fig. 3a,b, right). This is indicative of simultaneous operation of the acetyl-CoA and lysine degradation pathways (Fig. 2) and explained the formation of minor amounts of [2-^13^C]acetate, [2-^13^C]butyrate and [2,4-^13^C]butyrate in L-[6-^13^C]lysine. Altogether, these data provide molecular evidence that the lysine pathway was substantially active and generated the intermediates for the acetyl-CoA pathway in *Intestinimonas* AF211. Similar results supporting the simultaneous operation of the two pathways were obtained by using D,L-[2-^13^C]lysine in growing cells of *Intestinimonas* AF211, and the NMR analysis also confirmed the exclusive selectivity for L-lysine (Supplementary Fig. 2). High-performance liquid chromatography (HPLC) analysis confirmed the nearly complete conversion of lysine to equimolar amounts of butyrate and acetate as indicated above (Fig. 1a). The molecular events that explain the observed isotopomers derived from [^13^C]lysine were reconstructed (Supplementary Fig. 3) and the deduced metabolic pathway revealed 10 enzymatic reactions that were further characterized by genomic, proteomic and enzyme studies (Fig. 2).

### Proteogenomic analysis of the metabolic pathways

The complete genome of *Intestinimonas* AF211 was determined using single-molecule next-generation sequencing revealing a circular chromosome of 3,376,476 bp, predicted to encode 3,359 genes (NCBI accession number CP011307; to be reported elsewhere). Gene candidates for the fructoselysine and lysine metabolic pathways and linked energy-generating conversions were identified in the annotated genome of *Intestinimonas* AF211 (Fig. 2 and Supplementary Fig. 4). These included an operon-like cluster (AF_00949-00955) with genes for fructoselysine and psicoselysine uptake and degradation^14^ and a lysine utilization operon (AF_00976-00982) coding for six key enzymes involved in converting lysine into butyrate (Supplementary Fig. 4).

To confirm the presence and gene expression of the lysine degradation pathway in *Intestinimonas* AF211, proteins extracted from cells grown on either lysine or glucose and acetate were analysed by semi-quantitative proteomics (Supplementary Fig. 4 and Supplementary Data 1). *Intestinimonas* AF211 was found to produce all proteins involved in the conversion of glucose and acetate into butyrate and employed the acetyl-CoA pathway, similar to other members of Ruminococcaceae and Lachnospiraceae^8^. Moreover, the proteins involved in the lysine pathway (encoded by the cluster AF_00976-00982) were not only highly abundant but also differentially and ∼10-fold induced during growth on lysine compared with glucose and acetate, except for 3-aminobutyryl-CoA ammonia-lyase (encoded by AF_00976), which was not detected. The latter protein is rather small (14 kDa) and may have been lost during the protein extraction. Another annotated Kce candidate (AF_02981) was found to be reduced 10-fold during the growth in lysine and is present in very low level, pointing to another function rather than being involved in the lysine pathway. Two predicted 3-ketoacyl-CoA thiolases (AF_03338 and AF_00601) were 1.3-fold and 1.8-fold more abundant in lysine-grown cells than glucose and acetate-grown cells. AF_03338 is located in the same gene cluster operon with AtoD-A (AF_03339-03340; see below), which is a lysine-inducible key enzyme in the lysine pathway^19^. Thus, AF_03338 is likely to be co-expressed under lysine growth conditions, while the product of AF601 might participate in the simultaneous conversion of acetate into butyrate. Several predicted butyryl-CoA dehydrogenase (*bcd*) genes were detected in the genome but only one of these (AF_02889) formed an operon-like cluster with the Etf complex genes^25^ (AF_02890-02891). The reactions catalysed by ferredoxin oxidoreductase plus the exergonic NADH-dependent reduction of crotonyl-CoA to butyryl-CoA show a possible chemi-osmotic energy conservation via the Rnf complex^26^. This is essential under all growth conditions and hence the proteins encoded by this gene cluster were always detected (Fig. 2). The phosphate transacetylase (AF_00212) and acetate kinase (AF_01052) were found to be induced ∼3-fold in lysine-grown cells compared with glucose and acetate-grown cells, indicative of the involvement of these two enzymes in the lysine pathway. Remarkably, proteome analysis revealed the fructoselysine operon-like genes to be induced up to 25-fold during growth on lysine (Supplementary Fig. 4 and Supplementary Data 1), most likely due to a high amount of lysine, which was also an intermediate in the fructoselysine pathway (Fig. 2).

### Phylogeny of CoA transferases and enzyme activity

**Figure 4:**
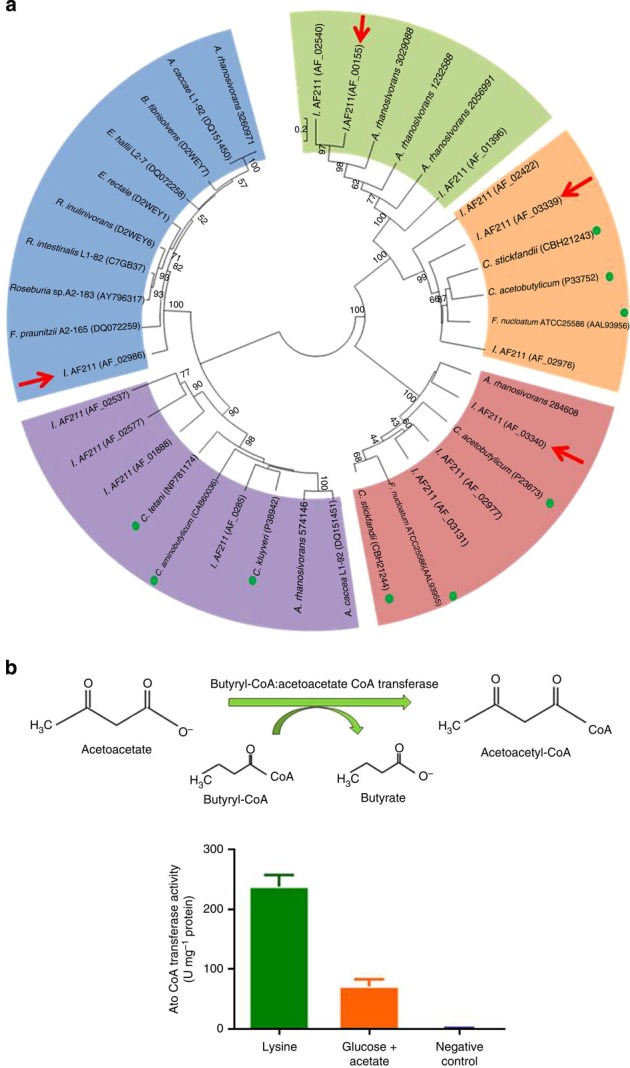
Phylogeny of CoA transferase and enzyme activity. (**a**) Phylogenetic tree of predicted CoA transferases from *Intestinimonas* AF211 (bold) and other representative anaerobes. The tree was based on sequences from butyryl-CoA:acetate CoA transferase (But, in blue), butyryl-CoA:4-hydroxybutyrate CoA transferase (4Hbt, in purple), butyryl-CoA:acetoacetate CoA transferase (Ato) alpha subunit (AtoD, in orange), beta subunit (AtoA, in brown) and acetyl-CoA:acetoacetate CoA transferase (AtoC, in green), respectively. Green dots indicate non-intestinal isolates. *Intestinimonas* AF211 proteins induced during growth on lysine are indicated by the red arrows. (**b**) Butyryl-CoA:acetoacetate CoA transferase activity in crude cell extracts. Each measurement was performed with biological duplicates and 4–6 replicate measurements. Values represent mean of replicates. Error bars indicate s.d.'s. Green and red bars represent the enzyme activity of *Intestinimonas* AF211 grown in lysine and glucose plus acetate, respectively; blue bar is the negative control with *A. rhamnosivorans* DSM26241 grown on glucose, which is not capable of lysine fermentation.

A crucial reaction involved in butyrate formation from lysine is the transfer of a CoA moiety from one molecule to another, catalysed by members of the CoA transferase family. Remarkably, analysis of the genome of *Intestinimonas* AF211 predicted the presence of 14 such enzymes, and a phylogenetic tree was generated based on these and CoA transferases from intestinal bacteria and environmental isolates (Fig. 4a). These include the CoA transferases AtoD-A, 4Hbt and But, for which experimental evidence for their involvement in butyrate synthesis has been described in several anaerobes, including *Clostridium SB4*, *Roseburia* spp., *Faecalibacterium prausnitzii* and *Clostridum acetobutylicum*^8^,^19^,^27^. Five well-separated clades of enzymes could be distinguished. One clade included the But enzymes from the main butyrate producers in the human intestine, belonging to the *Lachnospiraceae*, and *Ruminococcaceae*^4^ that are all capable of butyrogenesis from glucose or lactate plus acetate^8^,^28^. The *Intestinimonas* AF211 enzyme encoded by AF_02986 belonged to this But clade and was detected in the proteome under both growth conditions, indicating that it participated in the acetyl-CoA pathway rather than 4-aminobutyrate or glutamate pathways. The second clade harboured the 4Hbt enzymes and included several predicted CoA transferases of *Intestinimonas* AF211 but none was detected at the protein level. The third and fourth clades held the α and β subunits of the AtoD-A enzymes, respectively, that are encoded by two juxtaposed genes in the well-studied anaerobes: *F. nucleatum*, *C. sticklandii* and *C. acetobutylicum*^29^,^30^,^31^,^32^,^33^. Within these two AtoD-A subunit clades, three gene pairs of *Intestinimonas* AF211 were clustered. However, only one of these, encoded by AF_03339-03340, was highly abundant and induced two-fold under lysine growth conditions, indicative of its involvement in butyrate formation via the lysine pathway (Fig. 2). A fifth cluster of acetyl-CoA:acetoacetate CoA transferases (AtoC) contained enzymes not only from *Intestinimonas* AF211 (indicated by AF_00155, AF_02540 and AF_01396) but also from *Anaerostipes rhamnosivorans*, which is capable of butyrogenesis from glucose as well as from acetate plus lactate^34^. The four-fold protein induction of AtoC (AF_00155) during growth on lysine indicated the involvement of this CoA transferase in lysine degradation. We propose its role to be the balancing of acetyl-CoA, released from the acetyl-CoA pathway and providing either additional acetoacetate or acetoacetyl-CoA, in line with the simultaneous activity of the lysine and acetyl-CoA pathway during butyrogenesis from lysine (see above).

To further provide support for the pivotal conversion catalysed by AtoD-A, its activity in *Intestinimonas* AF211 was studied by incubating cell-free extracts under anaerobic conditions with acetoacetate and butyryl-CoA, and monitoring the production of acetoacetyl-CoA^19^,^30^. We found reproducible and high activity of this enzyme activity in cells grown on lysine (237 units per mg protein), which was 3.5-fold reduced in cells grown with glucose and acetate (71 units per mg protein; Fig. 4b). As the observed activity induction coincided with the protein quantitation from the proteome analysis, we deduce that AtoD-A (encoded by AF_03339-03340) is probably the butyryl-CoA:acetoacetate CoA transferase involved in the butyrogenic lysine pathway.

### *Intestinimonas* and fructoselysine genes in the human gut

As metagenome analysis indicated that the lysine pathway has high abundance in the human intestine^13^, we studied the presence of *Intestinimonas* related to AF211 in a series of healthy subjects by using a specific 16S rRNA-based quantitative PCR (qPCR) (Supplementary Table 3). In 5 out of 10 subjects, 0.2–10% of the 16S rRNA sequences were derived from *Intestinimonas* spp. since all qPCR products showed the expected nucleotide sequence. From faecal DNA of the remaining five subjects, amplicons were generated, but sequence analysis showed four of them to derive from *Ruminococcus* spp. as their 16S rRNAs fortuitously amplified with the used primers, indicating it is not possible to correctly estimate the level of *Intestinimonas* spp. in these subjects (Supplementary Table 3). When analysing the deep metagenome information obtained in 65 subjects characterized in the Human Microbiome Project^35^, we could identify many of the genes for lysine degradation in over half of the subjects but the genes involved in fructoselysine degradation were only observed in half a dozen individuals as indicated by the presence of the key gene fructoselysine kinase (Supplementary Fig. 5).

The observed abundance and prevalence of *Intestinimonas* spp. level is in good agreement with the metagenome-predicted presence of the lysine degradation pathway^13^, suggesting that *Intestinimonas* is the key species converting lysine and fructoselysine into butyrate in the human gut. This is supported by the recent isolation of a similar but antibiotic-resistant strain from a healthy subject^36^. However, it is evident that not all humans have intestinal metagenomes that are equipped with genes for the conversion of fructoselysine, the major Amadori product.

## Discussion

Here we describe the isolation of a butyrate-producing bacterium, *Intestinimonas* AF211, abundantly present in the intestine of some humans. The bacterium can use fructoselysine, a key intermediate in the formation of AGEs, as sole carbon and energy source by converting this into mainly butyrate and NH_3_. The predicted fructoselysine pathway includes the simultaneous degradation of both lysine and sugar moiety. By determining the metabolic route of the ^13^C-labelled lysine conversion by NMR, in combination with enzyme measurements and proteogenomic analysis, the butyrogenic pathway from lysine was fully elucidated and also indicated the presence of the fructoselysine pathway in *Intestinimonas* AF211. The use of fructoselysine as carbon and energy source for butyrogenesis is unique, and *Intestinimonas* AF211 is the first intestinal bacterium to harbour the complete pathway for the conversion of lysine into butyrate (Fig. 1), previously predicted based on metagenomic data^13^. Lysine is an essential amino acid that is cleaved from dietary proteins by pancreatic trypsin, producing peptide chains with a C-terminal arginine or lysine residue^37^, The generated lysine-containing peptides can be utilized via the proteolytic activity of *Intestinimonas* AF211 as evidenced by its growth on different protein-derived substrates (Supplementary Table 4) and the several-fold induction of various aminopeptidases under lysine growth condition (Supplementary Table 5). Collectively, one can consider the butyrogenic conversion of lysine by *Intestinimonas* AF211 as a specific example of host–microbe interactions.

Degradation of L-lysine was previously observed in complex media by *F. nucleatum*, suggesting that this Gram-negative bacterium and potential pathogen can use L-lysine as energy source but its use as carbon source is not clear^16^,^32^,^38^. *C. sticklandii* uses L-lysine as an electron donor in the Stickland reaction and lysine was only degraded in the stationary phase when other amino acids were depleted^29^. Despite the fact that a few bacteria have been reported to degrade lysine to butyrate, recent (meta) genomic analysis, which included genomes from these isolates, indicated that none of the genomes analysed had genes for the entire pathway^13^. In addition, *E. coli* and *Bacillus subtilis* were found to be capable of degrading fructoselysine but none of them produces butyrate^14^. However, *Intestinimonas* AF211 contained all genes involved in degradation of fructoselysine to butyrate (Fig. 2) while proteomic analysis revealed that these genes were induced under the lysine growth conditions.

Remarkably and unlike other butyrogenic bacteria, *Intestinimonas* AF211 was found to contain over a dozen genes coding for CoA transferases (Fig. 4a). We hypothesize that this may help the bacterium to be more flexible to act on a broad range of substrates. The integrated analysis of the *Intestinimonas* AF211 genome, proteome and activity measurements revealed that a specific acetoacetyl-CoA transferase AtoD-A (AF_03339-03340) was abundantly expressed under lysine degradation conditions and hence predicted to be the key enzyme involved in butyrate synthesis. As this is the first described lysine pathway in the intestinal ecosystem, the lysine utilization operon and the identified AtoD-A genes may have application as markers for butyrogenesis from lysine.

An important corollary of the abundant presence of lysine-degrading butyrogenic *Intestinimonas* AF211 and relatives in the intestine of some humans is the fact that amino acids can serve as source of butyrate formation. Since butyrate has presumed health benefits as an energy source for colonocytes and is a vital molecule to maintain intestinal integrity, this may nuance suggestions that protein fermentation, notably in the distal colon, has a negative health impact^39^. Remarkably, we observed that *Intestinimonas* AF211 is also capable of growth on fructoselysine as sole carbon and energy source resulting in the conversion of fructoselysine into butyrate (Supplementary Table 1). The Amadori product fructoselysine is abundant in cooked foods and is formed via the non-enzymatic Maillard reaction of reducing sugars and amino acids during the heating process. As humans are unique in the consumption of cooked products at a large scale, it would be of interest to determine whether the fructoselysine gene cluster detected in *Intestinimonas* AF211 has been recently acquired and is not present in the gut microbes of other primates. The production of fructoselysine from cooked foods has various impacts, including the loss of essential amino acids and a reduced protein digestibility. Fructoselysine is key product leading to the formation of AGEs in the human body that have been associated with chronic diseases and development of diabetes complication^40^,^41^,^42^. Moreover, recent food interventions in mice showed the deleterious effect of AGEs on these and other diseases^43^. The butyrogenic conversion of fructoselysine by *Intestinimonas* AF211 illustrates the important role of this anaerobe in the human intestinal tract, whereas the observation that some but not all human carry genes for fructoselysine degradation indicates the potential for specific interventions with the newly discovered strain. In conclusion, our study underlines the need for cultivating novel microbes to get a comprehensive understanding of the intestinal metabolic processes and the beneficial effect on the human host. In addition, *Intestinimonas* AF211 and related bacteria may play an important role in the intestinal tract by maintaining protein balance and gut homeostasis.

## Methods

### Enrichment, isolation and growth

Strain *Intestinimonas* AF211 was isolated from the stool of a healthy adult. The faecal sample was enriched in an anaerobic bicarbonate-buffered mineral salt medium^44^ containing 40 mM lactate and 40 mM acetate as energy and carbon source. The head space was filled with CO_2_/N_2_ (1:4) at 1.5 atm and incubation was at 37 °C. Subsequently, the enrichment culture was transferred to reinforced clostridium medium (RCM, Difco) in serial dilutions and plated at least three times on RCM agar, which resulted in an axenic culture. The purity of the strain, designated as strain AF211, was confirmed by 16S rRNA gene sequencing and microscopy. The strain was routinely maintained in RCM medium at 37 °C. A phylogenetic tree of the 16S rRNA of *Intestinimonas* AF211 (GenBank accession JX273469) and closely related strains was constructed as described previously^34^ (Supplementary Fig. 1).

For physiological characterization, different amino acids and amino-acid derivatives were tested including 20 mM of L-lysine, fructoselysine (USBiological, USA), glutamate, glutamine, glycine, proline, arginine, aspartate and methionine, which were added into the bicarbonate-buffered medium from 1 M sterile anoxic stock solutions. For sugar utilization 20 mM glucose plus 20 mM acetate were added into the bicarbonate-buffered medium from 1 M stock solutions. The growth was determined via product formation by HPLC^34^ and optical density measurement by a spectrophotometer at a wavelength of 600 nm. Generation times were calculated employing Gompertz modelling^45^.

### Analytical methods

Lysine was quantified on a HPLC using a Polaris C18-A column (Agilent) running at 45 °C and an ultraviolet–visible detector at wavelength of 436 nm. Flow rate was 0.5 ml min^−1^. An eluent mobile phase consisted of 24 mM acetic acid:8% acetonitrile (pH 6.6) as solvent A and acetonitrile:2-propanol (60:40) as solvent B. The gradient elution was used from 95% eluent A and 5% eluent B to 25% eluent A and 75% eluent B for first 15 min. The column was subsequently washed with 100% eluent B for 7 min before the next sample injection. Norleucine (4 mM) was used as internal standard. The volatile fatty acid formation was measured on a Thermo Scientific Spectra HPLC system equipped with an Agilent Metacarb 67H 300 × 6.5-mm column kept at 37 °C and running with 10 mM arabinose as an eluent. The detector was a refractive index detector. The eluent flow was 0.8 ml min^−1^. Gas production was performed as previously described^46^. All analyses were performed in duplicate. Ammonium was determined using Spectroquant test kit according to the manufacture's instruction. Fructoselysine was separated by ion-exchange chromatography and quantified by post column reaction with ninhydrin using photometric detection at 570 nm (International Organization for Standardization (ISO) 13903).

### Nuclear magnetic resonance

Strain AF211 was cultivated in a bicarbonate-buffered medium containing 20 mM of [2-^13^C]L-lysine or [6-^13^C]L-lysine or lysine plus [2-^13^C]acetate. The growth conditions were as described above. ^13^C-labelled lysine was purchased from Campro Scientific (Veenendaal, The Netherlands). Samples were taken from an overnight culture and centrifuged at 10,000*g*. D_2_O (50 μl; 99.9 atom%, Sigma Aldrich) was added to the supernatants (0.5 ml) and subsequently transferred in NMR tubes (Campro Scientific). ^1^H-decoupled ^13^C-NMR spectra were recorded at a probe temperature of 300 K on a Bruker Avance-III-500 spectrometer located at the Wageningen NMR Centre (WNMRC), Wageningen, the Netherlands. Chemical shifts are expressed in p.p.m. relative to the C-6 of added [6-^13^C]lysine at 41.75 p.p.m., added [2-^13^C]lysine at 57.19 p.p.m., formed [2-^13^C]butyrate at 42.33 p.p.m., formed [4-^13^C]butyrate at 15.95 p.p.m. and formed [2-^13^C]acetate at 25.99 p.p.m. (Biological Magnetic Resonance Data Bank, http://www.bmrb.wisc.edu/metabolomics/metabolomics_standards). For the HBMC spectra 400 experiments of 8 scans were recorded resulting in a measuring time of 50 min, using a standard Bruker pulse sequence. The products were identified based on chemical shifts as compared with database as mentioned above. In the HMBC experiment no decoupling is used (Fig. 3b). Therefore, the single-bond couplings will result in double cross-peak split by the large single-bond coupling. The active coupling between 5 and 10 Hz of two- and three-bond couplings remains within the width of a cross-peak in a HMBC spectrum, resulting in single cross-peaks. When the carbon attached to the proton involved in a three-bond coupling is also enriched, this cross-peak will be split by the large single-bond coupling with this carbon. These splittings are visible as extra peaks next to the single cross-peaks between H-2 and C-4 and between H-4 and C-2 (arrows in the figures), indicating double enrichment of both C-2 and C-4. The calculation was shown in Fig. 3c. Both the split and non-split HMBC cross-peaks with C-4 at H-2 and with C-2 at H-4 were integrated. The integrals of the split and non-split cross-peaks at a single proton are comparable, since they are based on the same three-bond couplings and experience similar relaxation behaviour. The split cross-peaks of H-2 with C-4 and of H-4 with C-2 show a small difference due to sensitivity of the HMBC experiment for difference in relaxation and difference in coupling values. Since these split cross-peaks refer to the same population of [2,4-^13^C]butyrate, the factor needed to equalize them can also be used to correct the value of the non-split values, thus revealing the percentages of all three possible fractions of labelled butyrate.

### Proteomics

The protein abundances in cultures growing with different substrates were investigated with liquid chromatography–mass spectrometry/mass spectrometry. Strain AF211 was grown in triplicate in 500 ml bicarbonate-buffered medium containing with 20 mM lysine and 40 mM glucose plus 40 mM sodium acetate as carbon and energy sources. Yeast extract was supplemented in the culture of glucose plus acetate (2 g l^−1^). Cells were collected in the exponential phase by centrifugation at 10,000*g* at 4 °C for 20 min. Cell pellets were washed twice in 100 mM Tris-HCl (pH 7.5), 1 mM dithioerythreitol and suspended in 1 ml of SDT-lysis buffer, which contained 100 mM Tris-HCl (pH 7.5), 4% SDS and 0.1 M dithiotreitol. Protein extractions, separation, tryptic digestion and analysis were performed as described previously^47^. An *Intestinimonas* AF211 database downloaded from Uniprot (http://www.uniprot.org) was used together with a contaminant database that contains sequences of common contaminants; for instance, trypsin, keratin, bovine serum albumin. The proteomics result contained peptides and proteins with a false discovery rate of <1% and proteins with at least two identified peptides of which should be unique and one should be unmodified without any reversed hits. The normal logarithm was taken from protein label-free quantitation (LFQ; normalized with respect to the total amount of protein and all of its identified peptides) intensities. Zero ‘Log LFQ' values were replaced by a value of 5.4 (just below the lowest value) to make sensible ratio calculations possible. Relative protein quantitation of sample to control was done with Perseus 1.3.0.4 by applying a two sample *t*-test using the ‘LFQ intensity' columns obtained with false discovery rate set to 0.05 and S0 set to 1.

### Preparation of *Intestinimonas* AF211 cell extract

Strain AF211 was grown in 150 ml anaerobic bicarbonate-buffered medium containing 20 mM lysine or 40 mM glucose plus 40 mM acetate as carbon and energy sources. Cells of strain *A. rhamnosivorans* DSM26241 (ref. 34) grown in 150 ml anaerobic bicarbonate-buffered medium containing 20 mM glucose were used as a negative control of the enzyme assay. Cells were collected in the exponential phase by centrifugation at 10,000*g* at 4 °C for 20 min. Cell pellets were washed twice in an anaerobic buffer containing 100 mM Tris-HCl (pH7.5), 1 mM dithioerythreitol and suspended in 1 ml of the same buffer. Cells were disrupted by sonication 5 times × 30 s and the cell suspension was cooled on ice for 30 s in between. Finally, the suspension was centrifuged for 10 min at 8,000*g*. The cell-free extract was transferred to serum bottles, flushed with N_2_ and either stored at −20 °C or used directly for enzyme activity assays. All steps were performed in an anaerobic chamber with a N_2_/H_2_ (96:4; v/v) atmosphere, circulated over a palladium catalyst to eliminate traces of oxygen.

### Detection of acetoacetyl-CoA transferase activity

CoA transferase activity was determined using a spectrophotometric assay^19^ at 310 nm, 25 °C with 100 mM Tris-Cl (pH 8.1), 20 mM MgCl_2_ and 50 μM butyryl-CoA plus 10 mM lithium acetoacetate as substrates. For enzyme activity assay the formation of acetoacetyl-CoA (*E*_310nm_=15.1 mM^−1^ cm^−1^) from butyryl-CoA and acetoacetate was followed by measuring the increase in *A*_310nm_ by means of an absorbance-recording spectrophotometer. Total volume of the mixture was 1.0 ml. The activities were expressed as U mg^−1^ of total proteins (U=μmol min^−1^). All assays were done with biological duplicates and 4–6 replicate measurements were performed. Total protein was quantified using Qubit2.0 Fluorometer (Invitrogen) according to the manufacturer's instructions.

### Phylogenetic analysis of CoA transferase

For the construction of a CoA transferase phylogenetic tree, all butyryl-CoA:acetate CoA transferases (Ato) and butyryl-CoA:4-hydroxybutyrate CoA transferases (4Hbt) from known intestinal butyrate-producing bacteria according to refs. 28, 48 were retrieved from the NCBI database (Fig. 4). Selected 4-hydroxybutyrate CoA transferases from *Clostridium kluyveri*, *C. acetobutyricum* and *C. tetani* were also included^28^. Butyryl-CoA-acetoacetate CoA tranferases (Ato) from *C. sticklandii* DSM519, *F. nucleatum* ATCC25586 and *C. acetobutylicum* ATCC824 were collected from their genomes^29^,^33^,^49^. These CoA transferases are catalysing either butyrate or butanol formation. All amino-acid sequences of 4Hbt and AtoA/C/D from strain *Intestinimonas* AF211 were aligned with retrieved sequences using the CLUSTAL_X programme. A phylogenetic tree was constructed using the neighbour-joining algorithm by the MEGA 5 with 1,000 bootstraps to obtain confidence levels for the branches.

### Quantification of *Intestinimonas* in the human colon

Stool samples of 10 Dutch healthy volunteers at different age and gender were collected (Supplementary Table 3). Genomic DNA was isolated via the bead-beating protocol described previously^50^. The 16S rRNA sequences of phylogenetically related species were retrieved from GenBank (www.ncbi.nlm.nih.gov) and used to perform multiple alignments by using CLUSTALW. Primers for qPCR were designed using DNASTAR programme according to Walter *et al.*^51^ Primers were PFF590f: 5′-AAAACTATGGGCTCAACCCA-3′ and PFF702r: 5′-GTCAGTTAATGTCCAGCAGG-3′ to quantify *Intestinimonas* AF211, which resulted in a 100-bp amplicon. Total bacteria were quantified using BAC1396F and PROK1492R primer pairs^52^. The 16S rRNA gene of strain AF211 was used for optimizing temperature and primer concentration, and for making standard curves. The programme that was used to amplify the partial 16S rRNA gene of strain AF211 was as following: 95 °C for 5 min and 35 cycles consisting of 95 °C for 30 s, 56.7 °C for 10 s and 72 °C for 30 s; 95 °C for 1 min and 60 °C for 1 min and total bacteria as followed: 95 °C for 10 min or 95 °C for 20 s, 56.3 °C for 30 s and 72 °C for 30 s; 95 °C for 1 min and 60 °C for 1 min. Both were followed by a melting curve analysis. DNA copies were calculated from standard curves and subsequently used for the strain quantities.

### Metagenome analysis

The metagenomic protein sequence data from faecal samples of 65 Human Microbiome Project subjects were obtained from MG-RAST (http://metagenomics.anl.gov/). The following samples were included: SRR063550, SRR063552, SRR063553, SRR063555, SRR063556, SRR063558, SRR063561, SRR063562, SRR063564, SRR063565, SRR061730, SRR063567, SRR063568, SRR063570, SRR063571, SRR063573, SRR063574, SRR063576, SRR063577, SRR063579, SRR063580, SRR063538, SRR063582, SRR063585, SRR063586, SRR063588, SRR063589, SRR063802, SRR063899, SRR063900, SRR063902, SRR063903, SRR063540, SRR063905, SRR063906, SRR063908, SRR063909, SRR063539, SRR063542, SRR063545, SRR063548, SRR063551, SRR063554, SRR063541, SRR063557, SRR063560, SRR063563, SRR063566, SRR063569, SRR063572, SRR063575, SRR063578, SRR063581, SRR063584, SRR063543, SRR063587, SRR063801, SRR063898, SRR063901, SRR063904, SRR063907, SRR063544, SRR063546, SRR063547 and SRR063549.

AF211 butyrogenic pathway protein homologues were searched from the metagenomic data by using Usearch v. 8.0.1517 with settings usearch_global and id=0.6. The results were analysed in R v. 3.1.1 software environment (http://www.R-project.org)^53^.

## Additional information

**How to cite this article**: Bui, T.P.N. *et al.* Production of butyrate from lysine and the Amadori product fructoselysine by a human gut commensal. *Nat. Commun.* 6:10062 doi: 10.1038/ncomms10062 (2015).

## Supplementary Material

Supplementary Information Supplementary Figures 1-5 and Supplementary Tables 1-5

Supplementary Data 1 Proteomic analysis of *Intestinimonas* AF211 grown at lysine or glcose/acetate.
