# Supplementary material for: Production of butyrate from lysine and the Amadori product fructoselysine by a human gut commensal
Source: Nat Commun. 2015 Dec 1;6:10062. doi: 10.1038/ncomms10062 (PMC4697335; doi:10.1038/ncomms10062)
Supplement: Supplementary Information — Supplementary Figures 1-5 and Supplementary Tables 1-5 [file ncomms10062-s1.pdf]

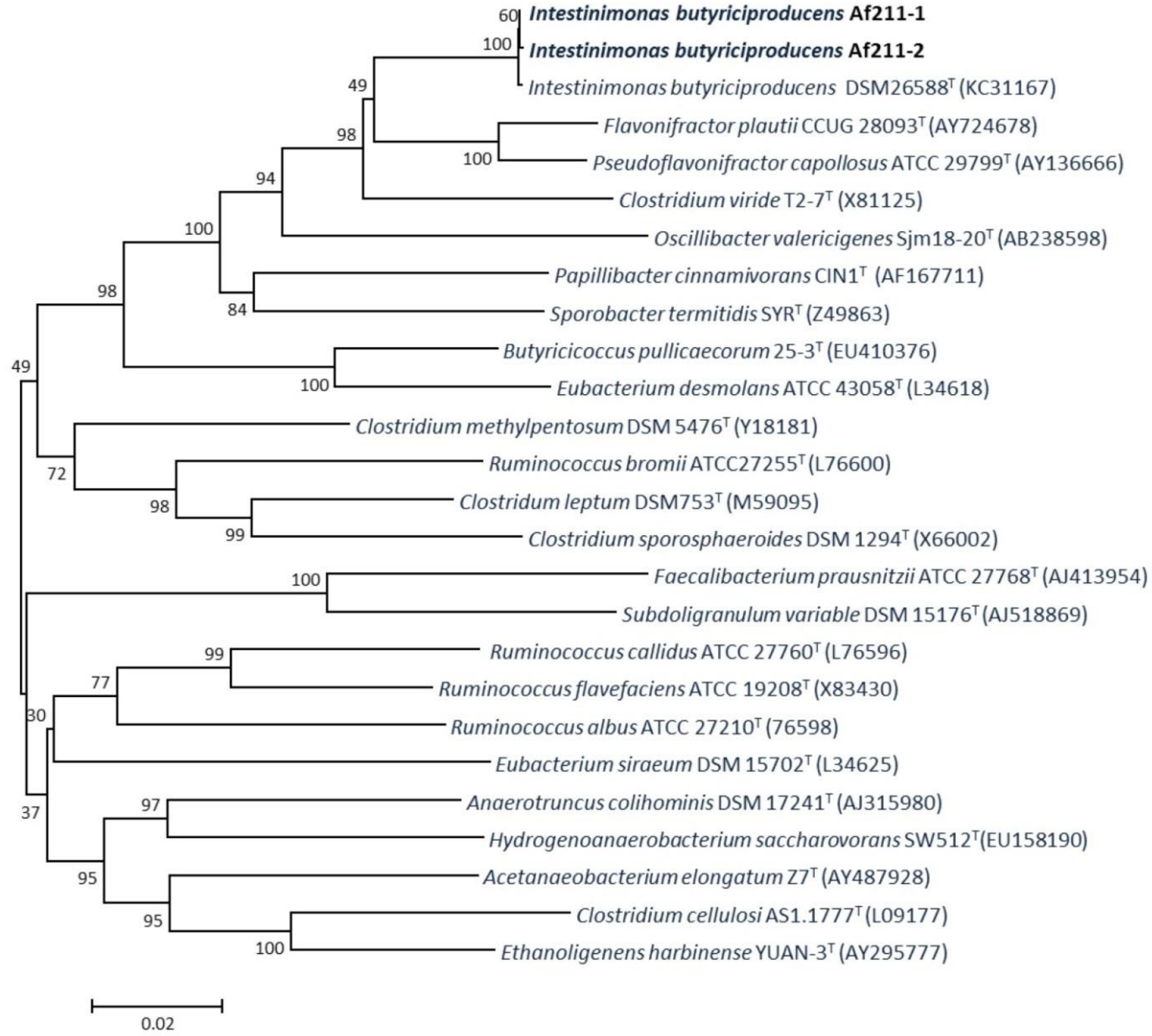

Supplementary Figure 1: Phylogenetic tree based on 16S rRNA gene sequences, showing the phylogenetic relation of strain AF211 and closely related members of *Clostridium* cluster IV. Bootstrap values > 50 % based on 1,000 replications are shown at branching points. Bar, 1 % sequence divergence.

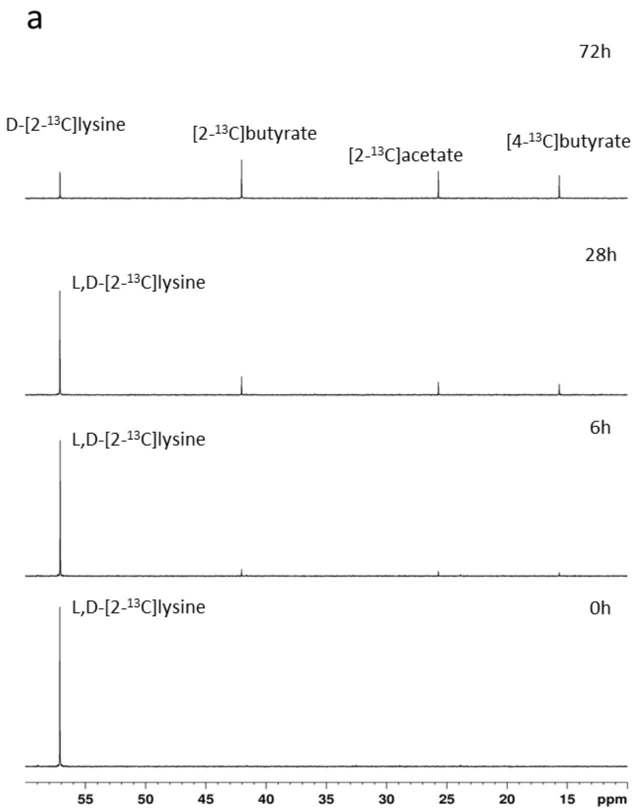

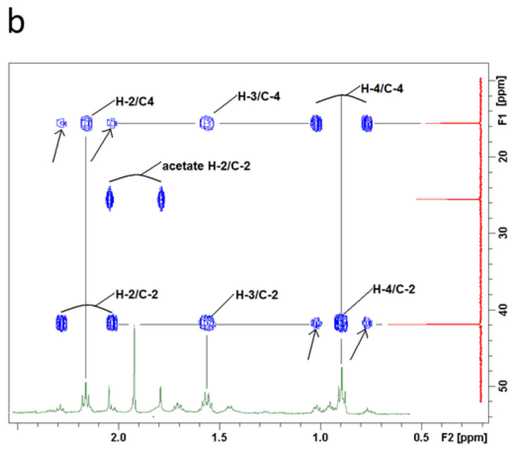

c

Product	Percentage (%)
[4-^{13}C]butyrate	23
[2-^{13}C]butyrate	59
[2,4-^{13}C]butyrate	17

Supplementary Figure 2. Elucidation of lysine pathway via ^{1}H-decoupled ^{13}C-NMR spectrum and 2D HMBC spectrum when grown in L,D-[2-^{13}C]lysine. a. High-resolution ^{1}H-decoupled ^{13}C-NMR spectra showing L,D-[2-^{13}C]lysine fermentation products. [2-^{13}C]butyrate, [2-^{13}C]acetate and [4-^{13}C]butyrate had a chemical shift of 42.33ppm, 25.99ppm and 15.95 ppm, respectively. **b**. 2D HMBC spectrum for L,D-[2-^{13}C]lysine is shown. **c**; Percentages of labelled butyrate fractions.

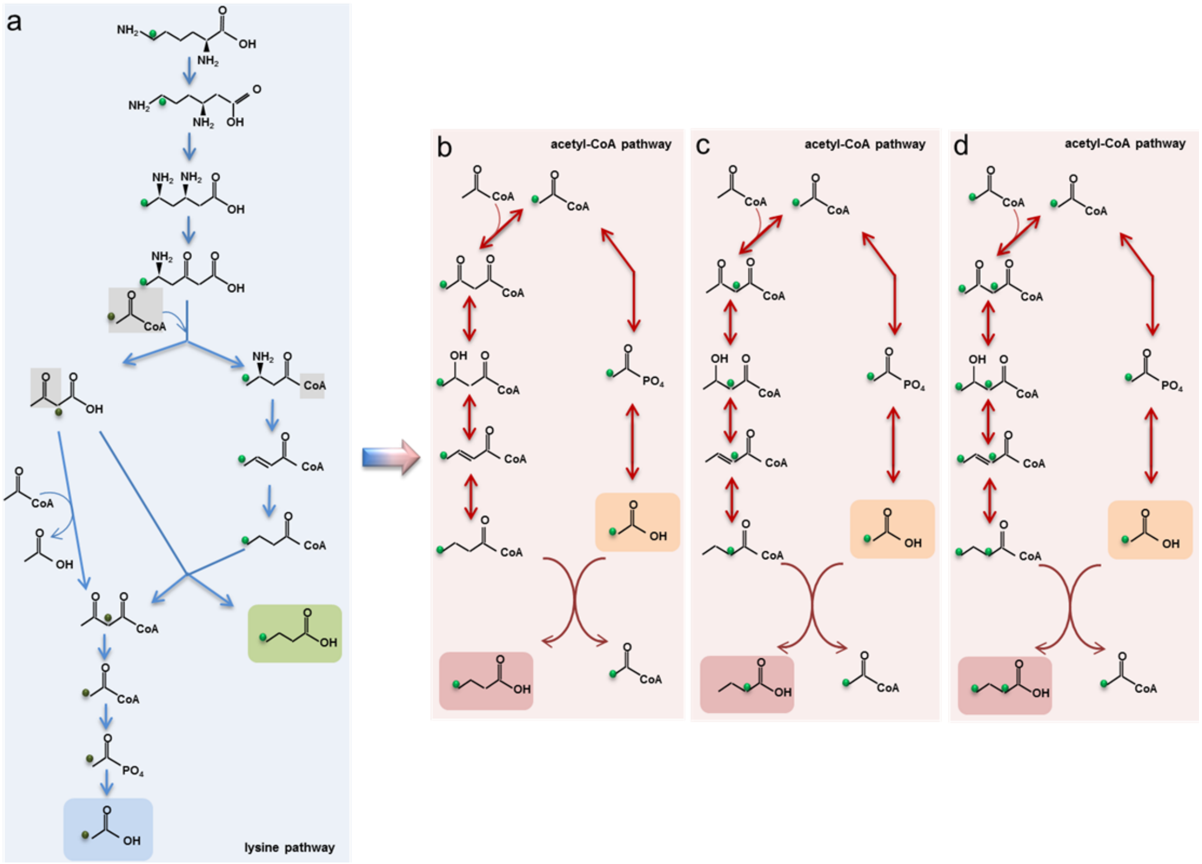

Supplementary Figure 3: The fate of ^{13}C-labelled carbon in *Intestinimonas* AF211. a: Proposed lysine pathway where L-[6-^{13}C]lysine resulted in [4-^{13}C]butyrate (in green). **b, c, d:** Acetyl-CoA pathway fed by intermediates of the lysine pathway. This pathway resulted in [2-^{13}C]butyrate, [4-^{13}C]butyrate, [2,4-^{13}C]butyrate and [2-^{13}C]acetate formation upon different combinations of either acetyl-CoA and [2-^{13}C]acetyl-CoA or 2 molecules of [2-^{13}C]acetyl-CoA. When labelled acetyl-CoA was plenty in the medium it also incorporated in the lysine pathway which ended up in [2-^{13}C]acetate production.

(a) Locus tag

Functions	Gene code	Locus tag	Fold induction
Uncharacterised protein	-	AF_00153	1.8
D-β-hydroxybutyrate permease	-	AF_00154	9.28
Acetyl-CoA:acetoacetate CoA transferase	AtoC	AF_00155	4.08
Phosphate acetyltransferase	Pta	AF_00212	3.25
L-Lysine permease	LysE	AF_00887	ND
Fructoselysine kinase	YhfQ	AF_00949	16.51
Fructoselysine 3-epimerase	YhfOP	AF_00950	ND
Fructosamine deglycase	YhfN	AF_00951	9.42
Spermidine putrescine ABC transporter permease component PotD	PotD	AF_00952	2.78
Spermidine putrescine ABC transporter permease component PotC	PotC	AF_00953	24.8
Spermidine putrescine ABC transporter permease component PotB	PotB	AF_00954	ND
Spermidine putrescine ABC transporter permease component PotA	PotA	AF_00955	20.21
3-aminobutyryl-CoA ammonia lyase	Kal	AF_00976	ND
3-keto-5-aminohexanoate cleavage enzyme	Kce	AF_00977	10.87
3,5-diaminobexanoate dehydrogenase	Kdd	AF_00979	7.07
Lysine 2,3-aminomutase	KamA	AF_00980	11.11
L-beta-lysine 5,6-aminomutase alpha subunit	KamD	AF_00981	6.25
L-beta-lysine 5,6-aminomutase beta subunit	KamE	AF_00982	11.26
Acetate kinase	Ack	AF_01052	2.83
Butyryl-CoA dehydrogenase	Bcd	AF_02889	0.93
Electron transfer flavoprotein α subunit	Etfα	AF_02890	0.60
Electron transfer flavoprotein β subunit	Etfβ	AF_02891	0.74
3-ketoacyl-CoA thiolase/Acetyl-CoA acetyltransferase	Thl	AF_03338	1.34
Butyrate-acetoacetate CoA-transferase α subunit	AtoD	AF_03339	1.57
Butyrate-acetoacetate CoA-transferase β subunit	AtoB	AF_03340	2.03
Proton pumping Rnf cluster (C, D, G, E, A, B subunit)	Rnf	AF_00682-00687	0.83; 0.83; ND (G,E,A); 0.92
V-type ATP synthase cluster (I, K, E, C, F, A, B, D subunit)	ATP synthase	AF_03050-03057	5.31; 308; 2.07; 5.01; -; 2.28; 2.53; 3.78
Inorganic Pyrophosphatase	Ppase	AF_02617	0.83
Ammonium transporter	NH_3 transporter	AF_00653, AF_01882	ND
Putative short chain fatty acids transporter (AF_01747, AF_02982, AF_03082, AF_03208)	SCFA trasporter	4 copies	0.15 (AF_01747)
Na+/H+ antiporter (AF_00191, AF_00924, AF_01158, AF_01159, AF_02156, AF_03116)	Antiporter	6 copies	0.56 (AF_01159); 0.3 (AF_03119)

(b) Gene arrangement

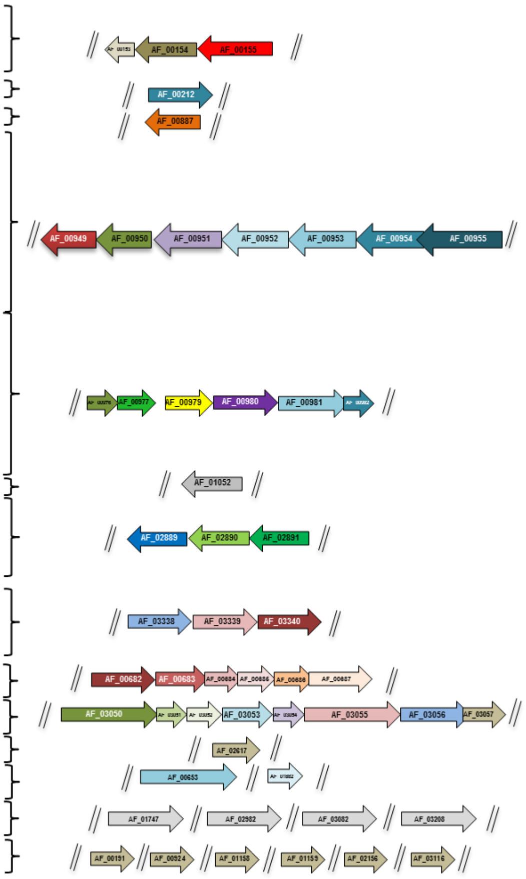

Supplementary Figure 4: Proteins involved in the fructoselysine and lysine conversion pathway (a) and their gene organization (b). The fold induction was deduced from the proteome analysis of *Intestinimonas* AF211 cells grown on lysine and GA (glucose plus acetate). ND: not detected.

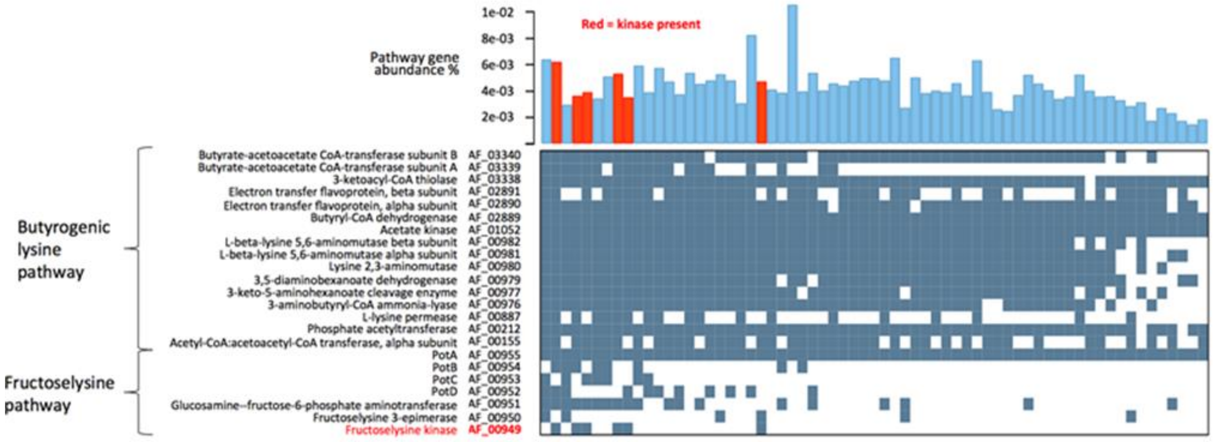

Supplementary Figure 5: Overview of butyrate pathway gene presence in Human Microbiome Project data. The matrix shows the presence (blue) and absence (white) of each gene in the two butyrogenic pathways in 65 HMP samples. The barplot shows the pathway gene numbers normalised by the read numbers in each sample. The red bars indicate the samples where sequences related to the fructoselysine kinase gene were found.

Supplementary Table 1: **Metabolites from glucose; lysine and fructoselysine fermentation.** Strain AF211 was grown in 17 mM L-lysine or 9 mM fructoselysine or 20 mM glucose plus 20 mM acetate. Values are means of duplicates ± standard deviation. The incubation time was 2 weeks for sugar fermentation, 2 days for lysine and 7 days for fructoselysine. ND: not detected. NA: not applicable. Carbon recovery data took into account the formation of CO_2. There was not any growth observed in D-lysine, glutamate, glutamine, glycine, proline, arginine, methionine and aspartate.

Substrate	Consumption (mM)		Production (mM)					Carbon recovery %	OD
	substrate	acetate	lactate	ethanol	acetate	butyrate	NH_4^+		
Fructoselysine	6.1 ± 1.0	0.29 ± 0.5	1.4 ± 0.2	ND	NA	14.9 ± 0.8	8.6 ± 0.5	101 ± 0.7	0.34
L-Lysine	16.8 ± 0.4	NA	ND	ND	15.6 ± 0.7	14.2 ± 0.6	22.1 ± 0.5	87 ± 2.5	0.36
Glucose	4.0 ± 0.2	2.8 ± 0.6	1.2 ± 0.5	1.7 ± 0.2	ND	4.4 ± 0.1	ND	83 ± 11.0	0.2

Supplementary Table 2: Acetate effect on fructoselysine growth. Strain *Intestinimonas* AF211 was grown in 4.3 mM fructoselysine without or with 10 mM acetate in duplicate. The incubation time was 6 days. ND: not detected. NA: not applicable. Carbon recovery data took into account the formation of CO_2. The OD values increased three-fold under all conditions but the growth rate were approximately 1.5 times higher on fructoselysine with acetate than on fructoselysine alone. The product balances also changed and without acetate, 1 fructoselysine was converted to 2 butyrate and 1 lactate while with acetate 1 fructoselysine was converted to 3 butyrate.

Substrate	Substrate consumption (mM)		Product (mM)				Carbon recovery %	Growth rate (h^{-1})
	Fructoselysine (added)	Acetate	Butyrate	Lactate	NH_4^+	Acetate		
Fructoselysine 1	4.3	NA	8.9	2.4	7.3	0.22	98 %	0.04
Fructoselysine 2	4.3	NA	8.5	2.9	9.4	0.5	96 %	
Fructoselysine-acetate 1	4.2	1.9	10.8	ND	6	NA	105 %	0.06
Fructoselysine-acetate 2	4.2	3.7	11.6	ND	7.9	NA	102 %	

Supplementary Table 3: Quantification of *Intestinimonas* AF211 in human fecal samples. A summary of the qPCR results and Sanger sequencing data is provided. Total DNA of *Intestinimonas* AF211 was amplified with 95% efficiency, compared to the 16S rRNA amplicon while total DNA of *Pseudoflavonifractor capilosus* or *Flavonifractor plautii* did not amplify with the *Intestinimonas* primers.

Volunteers	Total 16S copy number	*Intestinimonas* AF211 copy number	Percentage *Intestinimonas* (%)	Sequencing check
1	7.92E+04	2.67E+03	1.7	***Intestinimonas butyriciproducens***
2	1.80E+05	1.76E+04	4.9	*Ruminococcus bromi*
3	1.45E+05	1.07E+04	3.7	*Ruminococcus bromi*
4	1.98E+05	1.68E+04	4.2	No data
5	8.27E+04	2.50E+02	0.15	***Intestinimonas butyriciproducens***
6	2.54E+05	1.27E+03	0.25	***Intestinimonas butyriciproducens***
7	1.50E+05	2.95E+04	9.8	***Intestinimonas butyriciproducens***
8	6.15E+04	1.31E+03	1.1	***Intestinimonas butyriciproducens***
9	9.79E+04	1.63E+02	0.09	*Ruminococcus bromi*
10	6.34E+04	7.44E+03	2.4	*Ruminococcus bromi*
gDNA AF211	7.77E+05	7.43E+05	95.58	

Supplementary Table 4: Product formation in different protein sources. Short chain fatty acid production was analysed after a week growth in the bicarbonate buffered media plus 10g/l of tryptic soy broth without dextrose (BD), tryptone (BD), casitone (BD), vegetable peptone (BD), yeast extract (BD), bacterial peptone (BD), casein hydrolysate (BD), methyllysine (SIGMA), dimethyllysine (SIGMA) or hydroxylysine (SIGMA).

Substrates (10g/l)	Acetate (mM)	Propionate (mM)	Butyrate (mM)
Tryptic Soy Broth w/o dextrox	1.85	1.76	2.23
Tryptone	3.74	3.13	3.95
Casitone	4.52	0.81	3.46
Vegetable Peptone	1.09	0	0.55
Yeast Extract	3.20	3.04	5.04
Bacterial Peptone	0.81	0	0.70
Casein Hydrolysate	6.77	0.65	3.52
Methyllysine	0	0	0
Dimethyllysine	0	0	0
Hydroxylysine	0	0	0

Supplementary Table 5: Aminopeptidases found and detected from the whole proteome. The fold induction was deduced from the proteome analysis of *Intestinimonas* AF211 cells grown on lysine and GA (glucose plus acetate).

Functions	locus tags	Fold induction
Methionine aminopeptidase (EC 3.4.11.18)	AF_03023c	4.15
Tripeptide aminopeptidase (EC 3.4.11.4)	AF_00964	2.17
Aminopeptidase YpdF (MP-, MA-, MS-, AP-, NP- specific)	AF_01181	2.75
peptidase M18, aminopeptidase I	AF_01776c	3.09
Tripeptide aminopeptidase (EC 3.4.11.4)	AF_02694	7.03
Deblocking aminopeptidase (EC 3.4.11.-)	AF_01656c	-1.46
Deblocking aminopeptidase (EC 3.4.11.-)	AF_01657c	1.19
Deblocking aminopeptidase (EC 3.4.11.-)	AF_01658c	-1.07
